# The novel features of *Plantagoovata* seed mucilage accumulation, storage and release

**DOI:** 10.1038/s41598-020-68685-w

**Published:** 2020-07-16

**Authors:** Jana L. Phan, James M. Cowley, Kylie A. Neumann, Lina Herliana, Lisa A. O’Donovan, Rachel A. Burton

**Affiliations:** 1 Australian Research Council Centre of Excellence in Plant Cell Walls, School of Agriculture, Food and Wine, University of Adelaide, Waite Campus, Urrbrae, SA 5064 Australia; 2 Australian Research Council Centre of Excellence in Plant Energy Biology, School of Agriculture, Food and Wine, University of Adelaide, Waite Campus, Urrbrae, SA 5064 Australia; 3 Present Address: Australian Academy of Science, Ian Potter House, 9 Gordon St, Canberra, ACT 2601 Australia; 4 Present Address: IP Australia, PO Box 200, Woden, ACT 2606 Australia

**Keywords:** Cell wall, Plant physiology

## Abstract

Seed mucilage polysaccharide production, storage and release in *Plantago* *ovata* is strikingly different to that of the model plant *Arabidopsis*. We have used microscopy techniques to track the development of mucilage secretory cells and demonstrate that mature *P.* *ovata* seeds do not have an outer intact cell layer within which the polysaccharides surround internal columellae. Instead, dehydrated mucilage is spread in a thin homogenous layer over the entire seed surface and upon wetting expands directly outwards, away from the seed. Observing mucilage expansion in real time combined with compositional analysis allowed mucilage layer definition and the roles they play in mucilage release and architecture upon hydration to be explored. The first emergent layer of hydrated mucilage is rich in pectin, extremely hydrophilic, and forms an expansion front that functions to ‘jumpstart’ hydration and swelling of the second layer. This next layer, comprising the bulk of the expanded seed mucilage, is predominantly composed of heteroxylan and appears to provide much of the structural integrity. Our results indicate that the synthesis, deposition, desiccation, and final storage position of mucilage polysaccharides must be carefully orchestrated, although many of these processes are not yet fully defined and vary widely between myxospermous plant species.

## Introduction

Upon exposure to aqueous environments, seeds from myxospermous species extrude a polysaccharide-rich gel from their seed surface, often called mucilage. Numerous species display myxospermy and there are a range of possible evolutionary advantages of synthesising such a carbon-rich and energy-expensive substance^1^. Of all myxospermous species, the seed mucilage system of *Arabidopsis* is the best characterised. *Arabidopsis* seed mucilage has been used extensively as a proxy for the study of plant cell wall polysaccharide biosynthesis, enabling increased molecular characterisation of pectin biosynthesis, its main polysaccharide component^2^, as well as the biosynthesis of cellulose^3,4^ and several hemicelluloses^5–8^, which are minor but integral components*.* Mucilage from other species can be highly diverse^1^ and while *P.* *ovata* mucilage is also a complex mixture of polymers, it is predominantly heteroxylan with only a minor pectin component. While the pectin component is a near-linear rhamnogalacturonan^9–11^, the *P.* *ovata* heteroxylan (accounting for around 90% of the mucilage polysaccharides) is highly complex with the current scientific consensus defining *P.* *ovata* heteroxylan comprising a β-(1,4)-linked-d-xylopyranose backbone, heavily substituted at O-2 and/or O-3 positions with various mono-, di- and oligosaccharide substitutions of α-l-arabinofuranose and β-d-xylopyranose^9,11,12^. It is likely that, as with other eudicots, the β-(1,4)-linked-d-xylopyranose backbone is synthesised by several members of glycosyltransferase (GT) families 43 and 47. There is strong evidence that GT47 protein IRX10-L, probably in concert with other GT43 and GT47 proteins, extends the backbone by adding UDP-xylose moieties^14,15^. GT61 family members have been implicated in both α-arabinosyltransferase and β-xylosyltransferase xylan backbone decoration activities in cereals^16,17^, *Arabidopsis*^18,19^ and *Plantago*^20^*,* and copy number and type of GT61 was found to influence interspecific differences in *Plantago* heteroxylan fine structure^11^. The overall picture is complicated even further in that different fractions (sometimes described as layers) of *P.* *ovata* mucilage contain heteroxylans of varying substitution patterns showing that, like *Arabidopsis* mucilage, it is a similarly complex but orchestrated network of polysaccharides^9,10,13^. To date, many xylan synthase genes, particularly those involved in backbone decoration, are still unknown.

*Plantago* *ovata* mucilage also has economic relevance, in that in its dry state it constitutes the basis of a dietary fibre supplement, called psyllium, that is widely consumed by humans to assist with laxation, relieving constipation^21,22^, and to treat metabolic disorders like hypercholesterolaemia^23^. More recently, psyllium has become a key ingredient in gluten-free food, where it provides texture and structure in the absence of gluten^24–27^. Psyllium is produced by milling the dry polysaccharides off the seed surface^28^ and is often referred to as the “husk” fraction. The ratio of husk to seed is approximately 1:3, with the discarded non-husk seed components often being used for animal or fish feed^29^. From an economic standpoint, the ability to understand mucilage polysaccharide production and therefore potentially increase the valuable husk fraction is a viable breeding target for this plant species.

As well as studying the biosynthesis of mucilaginous polymers, the mechanism of mucilage extrusion from the seed coat of *Arabidopsis* has also been thoroughly characterised. In *Arabidopsis*, seed mucilage polysaccharides accumulate in the apoplast of specialised seed coat cells called ‘mucilage secretory cells’ (MSCs). When the mucilage polysaccharides become hydrated, they swell and rupture the primary cell wall of the MSC, releasing the mucilage^30^. The MSCs in *Arabidopsis* differentiate from the outer-most integument cell layer of the ovule. *Arabidopsis* has an outer integument composed of two cell layers and an inner integument composed of three cell layers, both of maternal origin, which grow to surround the mature ovule^31^. After pollination, at approximately 7 days post-anthesis (DPA), starch granules begin to accumulate in the MSCs and polysaccharide deposition starts in the peripheral “corners” of the cells. This pushes the protoplasm to form a central volcano-like structure in the cell. At 10 DPA this central column is reinforced by the deposition of secondary cell wall polysaccharides to form the columella. The columella is a prominent feature of the mature MSCs in *Arabidopsis* and the accumulated polysaccharides are deposited and stored around it, producing a doughnut-shaped ring. This structure results in the distinctive mature *Arabidopsis* seed coat patterning seen using SEM^32–34^. The details of MSC development, rupture and mucilage release are discussed in comprehensive reviews by Francoz et al*.*^30^, and Voiniciuc et al*.*^35^. An important developmental stage during MSC development is the weakening of the radial primary cell walls of the MSCs at the end of columella formation, at approximately 13 DPA. This process enables the consequent fracturing and rupturing of the cell walls of the MSCs upon imbibition in an aqueous environment^32^. The rupturing allows the accumulated seed polysaccharides to be extruded almost instantaneously forming the distinctive mucilage envelope. Thus, MSCs of *Arabidopsis* are a highly-specialised seed coat cell with a clearly defined structure that is essential for correct seed mucilage extrusion. MSC development and mucilage release of *Linum* *usitatissimum* seeds, more commonly known as flax, has also recently been described, revealing an even more complex MSC structure^36^. The flax MSCs, embedded in the external surface of the seed coat were determined by Miart et al*.*^36^, to contain four discrete, laminated layers in the apoplast, each containing chemically- and functionally-distinct polysaccharides. Each of the layers and their polysaccharide contents act in concert to effectively hydrate the polysaccharides, mechanically forcing the radial cell wall to rupture in a peeling fashion and enabling mucilage to be released. In other species such as *Salvia* *hispanica* (chia) and *Coleus* *blumei*, the seed mucilage polysaccharides are stored in the outer epidermal cell layer(s) of a nutlet that encases the true seed within^37,38^, making these species myxocarpous rather than myxospermous. The events leading to the release of mucilage in these species have not been documented in detail but there appears to be great diversity in the mucilage extrusion structures between plant types^1^.

The accumulation of seed mucilage polysaccharides in *P.* *ovata* has been investigated previously^39^ and appears to be distinct from the process observed in the *Arabidopsis* MSCs. In the case of *P.* *ovata*, seed mucilage polysaccharides are deposited in the outer-most cell layer of a large integument. This single cell layer accumulates polysaccharides rapidly and the cells expand dramatically in size in a process that does not involve the formation of a central columella^39^. Beyond this, little is known about the precise development of these cells and so here we provide a detailed characterisation of the polysaccharide deposition and mucilage release processes of *P.* *ovata*, also enabling the formulation of a supporting model.

## Results

### Development of *P. ovata* mucilage secretory cells

**Figure 1:**
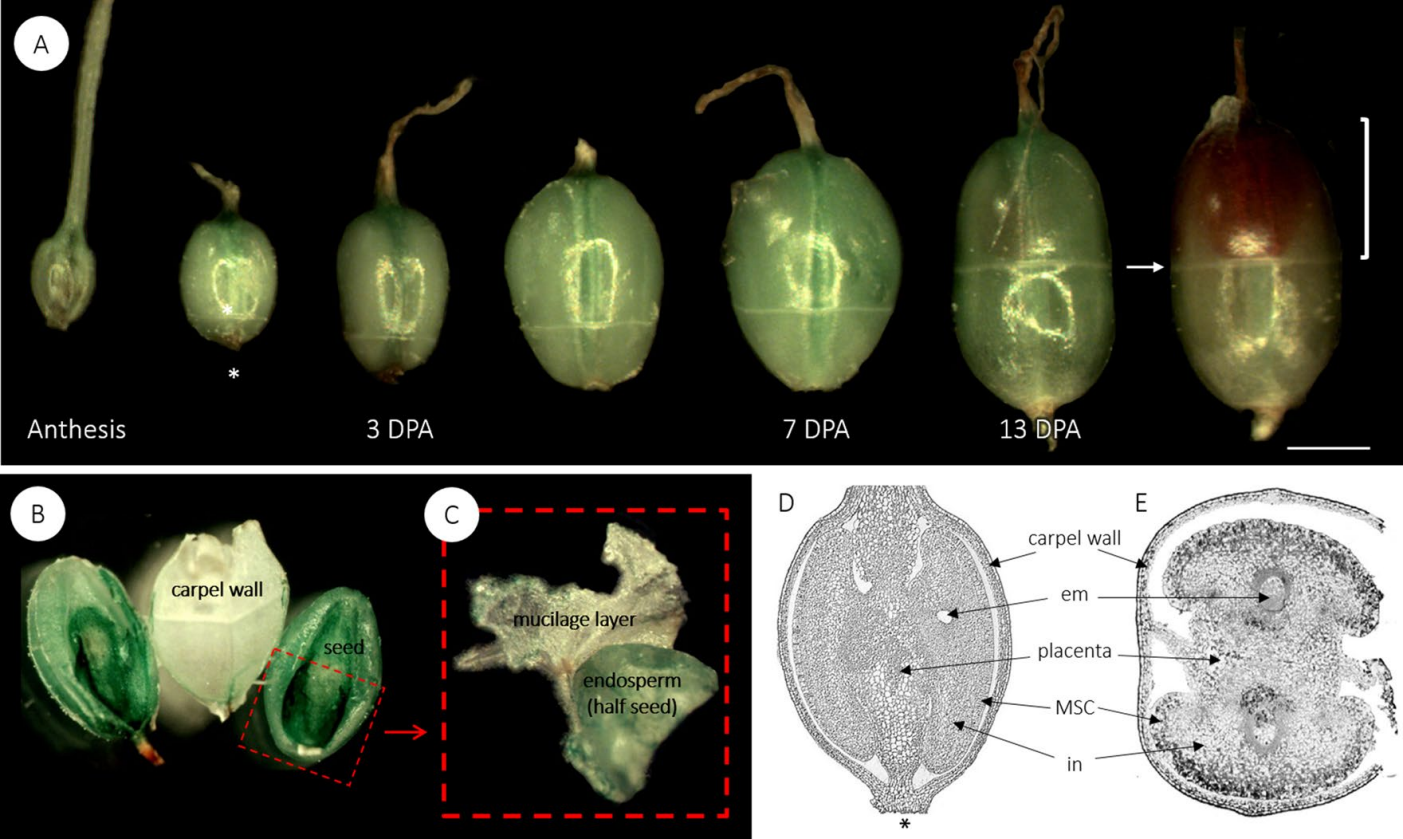
(**A**) The fruit development of *P.* *ovata*. Each fruit contains two ovules separated by placental tissue. *Plantago* species have a circumscissile capsule, also known as a pyxis. The arrow indicates the equator, where the zone of dehiscence is visible, the square bracket highlights the operculum, which detaches during dehiscence, and *indicates the end that joins the fruit to the rachis. Bar 1 mm. (**B**) A dissected fruit at 13 DPA, showing two immature seeds and in (**C**) one of the seeds has been further dissected to show the mucilage polysaccharide layer, which has been peeled off the seed and is the remnant of the integument tissue. Longitudinal (**D**) and transverse (**E**) cross sections of a developing fruit at 7 DPA, stained with toluidine blue. *Em* embryo sac, *MSC* mucilage secretory cells, *in* integument.

*P. ovata* takes approximately 3.5 months to grow from germination through to maturity. The mature plants have long slender, straggly leaves and produce many spike-type infloresences (SI Fig. S1). Development of *P.* *ovata* fruit on the spike and length of the inflorescence (and consequently yield per plant) are strongly dependent on the plant’s health during growth and development. Each fruit or capsule contains two ovules, separated by a maternal disc and joined to the parent plant via a placenta (Fig. 1). *P.* *ovata* possesses a circumscissile capsule (also called a pyxis) that is firmly attached to the inflorescence at the proximal end of the fruit. When the fruit is mature, the seed dispersal mechanism involves dehiscence at the capsule equator causing the operculum to detach, enabling the seed to dislodge from the capsule. The operculum, the point of attachment to the rachis, and the equator are indicated in Fig. 1A. After pollination, the fruits mature in approximately 1 month. At 2 weeks post-anthesis, the fruit has reached its full length and the seeds continue to develop inside, expanding widthways and filling the fruit.

**Figure 2:**
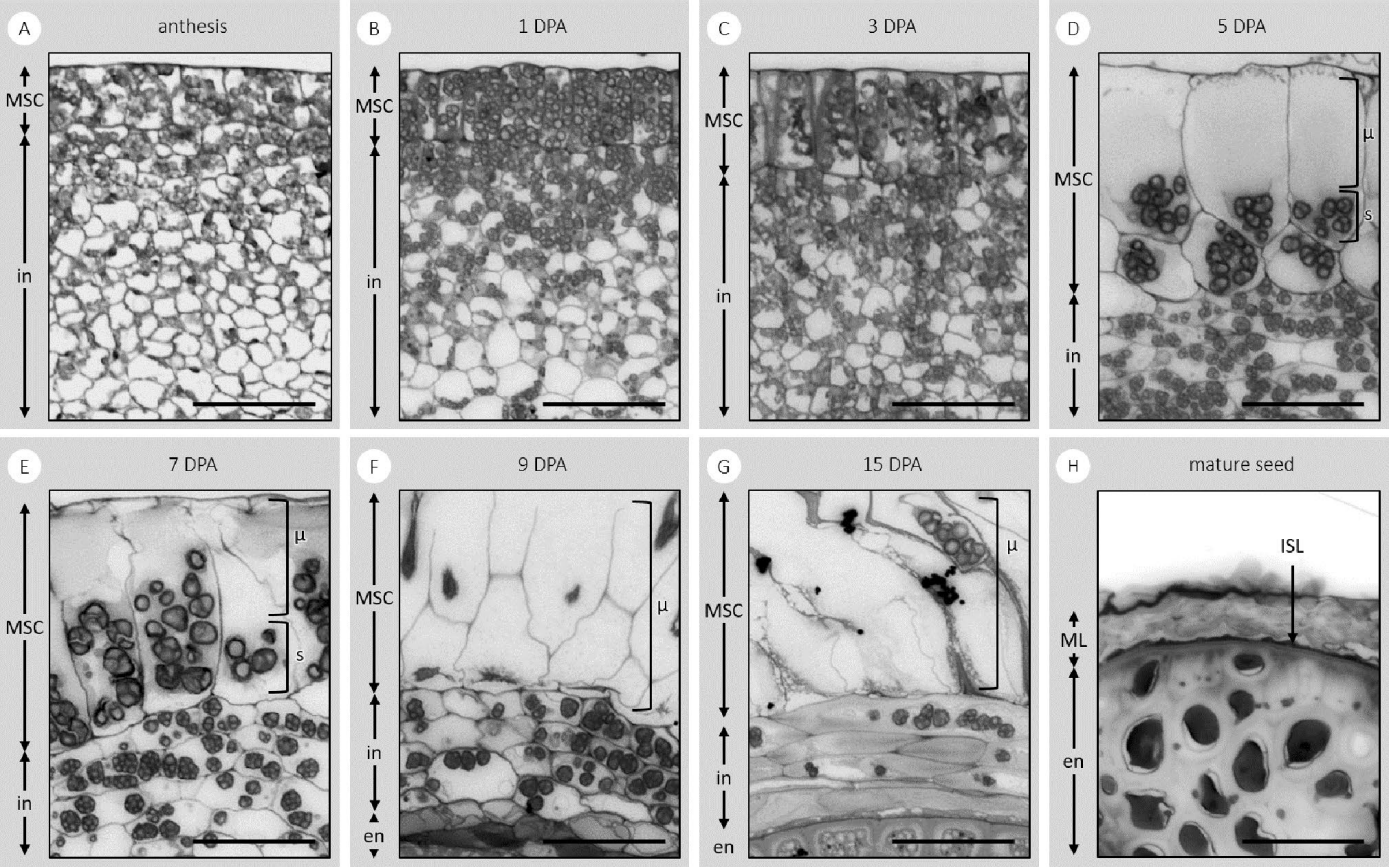
Toluidine blue-stained transverse sections of the developing integument of *P.* *ovata*. The sections show the tissues and components that are the: endosperm (en); integument (in); mucilage polysaccharides (μ); mucilage secretory cells (MSCs); mucilage polysaccharide layer (ML); intensely stained layer (ISL); and starch granules (s) at days post-anthesis (DPA). Scale bar 50 μm.

Following successful fertilisation, the parenchyma cells of the integument layers differentiate rapidly (Fig. 2). The MSCs of *P.* *ovata* seeds are easily observed at 1 DPA. They develop from the outermost single cell layer of the integument and lengthen as they accumulate starch granules (Fig. 2B,C). Substantial growth and elongation of the MSCs is observed from 3 to 5 DPA. Although it is difficult to discern discrete cellular compartments, it is likely that the empty space at the distal end of the cells where the polysaccharides accumulate, is the apoplast (Fig. 2D). By 9 DPA, the accumulated mucilage polysaccharides are hydrophilic enough to rupture the MSCs when they come into contact with aqueous solutions and it is technically challenging to obtain intact sections from this stage onwards. At 15 DPA all MSCs have ruptured and released their mucilage in fixed and sectioned developing seeds but it is possible to observe that the integument has been compressed to just a few cell layers between the MSCs and the seed endosperm. This compressed layer has disappeared almost completely by the time the seed is fully mature, leaving only a thin layer situated between the endosperm and the mucilage polysaccharide layer (Fig. 2H).

### Composition of developing ovule cell walls

**Figure 3:**
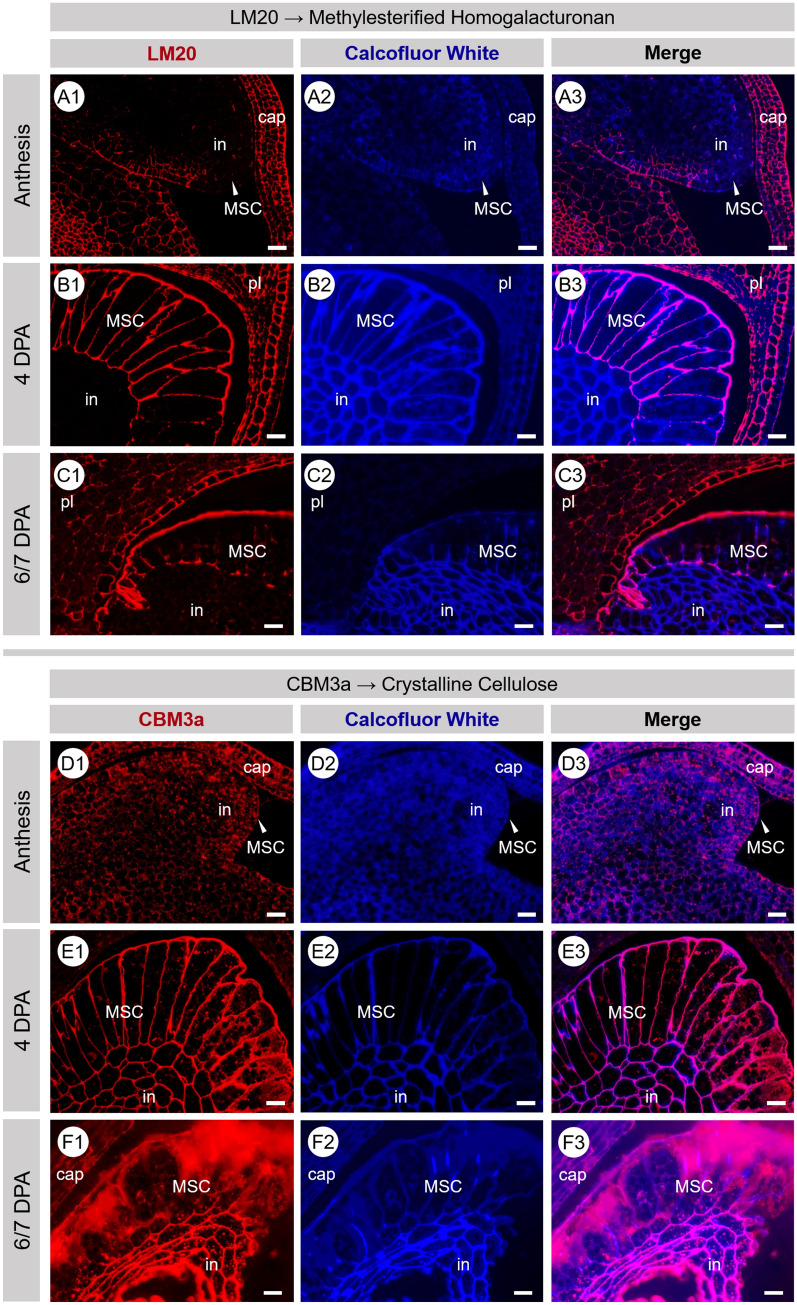
Fluorescence micrographs of transverse sections of developing *P.* *ovata* seeds labelled with LM20 and CBM3a (red/pink) at anthesis (**A**,**D**), at 4 DPA (**B**,**E**) and at 6/7 DPA (**C**,**F**). MSC cell walls show strong labelling of highly methylesterified HG (LM20) and crystalline cellulose (CBM3a) that diminishes in intensity and organisation as development/mucilage polysaccharide accumulation continues and/or as MSC cell walls disintegrate. Samples are counter-stained with calcofluor white (blue). Scale 20 μm. *DPA* days post-anthesis, *HG* homogalacturonan, *MSC* mucilage secretory cell, *in* integument, *pl* placenta, *cap* capsule.

The cell walls of seed tissues at three key time points across early development were fluorescently immunolabelled with the primary antibodies LM11 (β-1,4-linked xylan backbone^40^) LM19 (un-esterified and partially esterified homogalacturonan^41^) and LM20 (methylesterified homogalacturonan^41^) and the carbohydrate-binding module CBM3a (crystalline cellulose^42^). At anthesis the ovule is minute but there was clear binding by both LM20 (Fig. 3A1–3) and LM19 (SI Fig. S2A), with the latter producing a strong signal in the integument tissue. There was a low level of CBM3a binding to the walls of both MSCs and integument cells (Fig. 3D1–3) and no binding by LM11 (SI Fig. S2B). At 4 DPA, the MSCs are greatly elongated. The strongest signals are generated by LM20 in the MSC layer (Fig. 3B1–3) and CBM3a in both the MSC and integument cells (Fig. 3E1–3) but there was no labelling evident for LM19 (SI Fig. S2C) or LM11 (SI Fig. S2D). The final time point was at 6/7 DPA when the MSCs were becoming fragile due to the accumulation of mucilage polysaccharides. At this point the LM20 labelling was now restricted to the outside edge of the MSC layer and in cell corners bordering the integument tissue (Fig. 3C1–3). The CBM3a signal was still present in both the MSCs and integument though signals in the MSCs had become non-specific and amorphous compared to the integument where labelling of distinct walls was still present (Fig. 3F1–3). By 6/7 DPA there was no labelling by LM19 (SI Fig. S2E) and minimal labelling by LM11 (SI Fig. S2F).

### Surface features of mature *P. ovata* seeds

**Figure 4:**
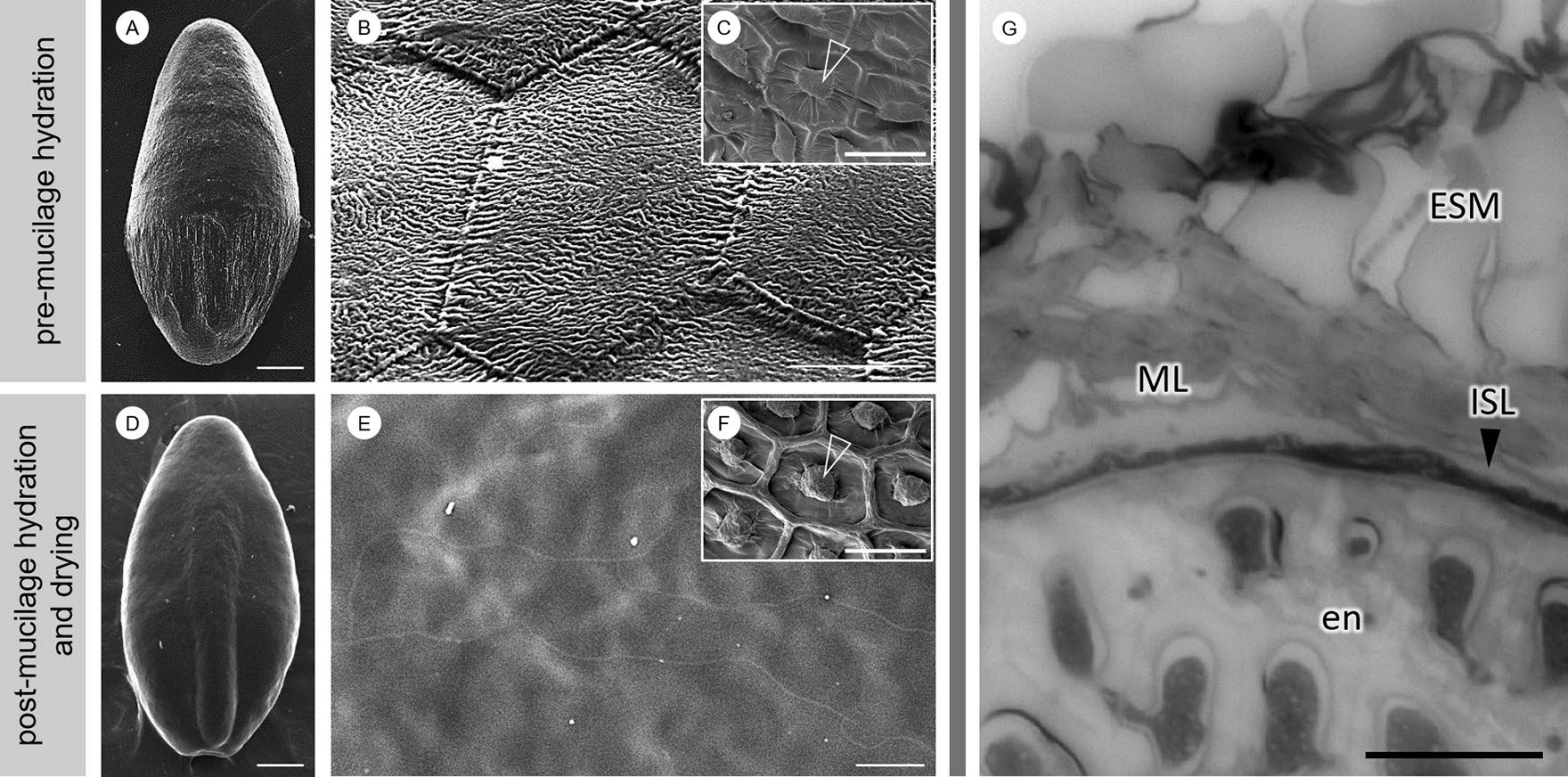
Scanning electron micrographs show that *P.* *ovata* does not contain a columella (**A**,**B**). Inset (**C**) shows a scanning electron micrograph of the seed surface of *Arabidopsis* with the columella structure indicated with an arrowhead. In *P.* *ovata,* the wrinkled texture of the dry mucilage polysaccharide layer (ML) and hexagonal shapes of the distal MSC wall remnants disappear after mucilage is hydrated and allowed to dry back onto the seed surface, unfixed (**D**,**E**), leaving it extremely smooth. This contrasts with *Arabidopsis* where the distinct columella structure persists and remains clearly visible after the same process (**F**). Toluidine blue-stained cross sections of the mature seeds fixed in aqueous fixative (**G**) reveal that the seed mucilage (ESM) expands from the ML, which sits on top of an intensely stained layer (ISL) that separates the mucilage polysaccharide layer from the endosperm. *En* endosperm. Scales **A**,**D**,**G** 500 μm; **B**,**E**,**H** 50 μm; **C**,**F** 30 μm.

The mature seeds of *P.* *ovata* have a deep scar on the ventral side resulting in a boat-shaped seed (SI Fig. S3). The patterning of the dry seed surface on the dorsal side is polar. Where the inner surface of the fruit capsule has been pressed against the seed it is smoother (Fig. 4A). The seed surface is covered in hexagonal structures with a distinct wrinkled texture (Fig. 4B). The wrinkled patterning and hexagonal structures on the mature seed surface are lost once seeds have been imbibed in water (Fig. 4D). When the remaining seed mucilage is left to dry back onto the seed after hydration in cold water, the seed surface appears very smooth and high magnification SEM reveals little additional detail (Fig. 4E). The hexagonal structures are no longer visible, and the polarity observed prior to mucilage expansion (Fig. 4A) has also been lost. This is in clear contrast to *Arabidopsis* where, after the same process, the seed surface morphology remains relatively unchanged and the columella is still clearly visible (Fig. 4F). Mature *P.* *ovata* seeds therefore do not have conventional seed coat cells and there is certainly no columella as found in *Arabidopsis* (Fig. 4C). Rather there is a dehydrated mucilage polysaccharide layer, underlain by a thin dark brown layer (which gives the seed its colour) both of which sit over the outer layer of the endosperm (Fig. 4G). The crushed integument layer is not at all visible in the mature seed.

### Mucilage removal from mature *P. ovata* seeds

Different methods were used to remove the expanded mucilage from mature imbibed seeds of *P.* *ovata*. Although previous studies report no significant compositional differences between the different extraction methods^11^, some physical differences were observed on the exposed seed surface (SI Fig. S4). When mature seeds were placed into an aqueous fixative, the sequential washing steps removed most of the mucilage from the seed, leaving a thin resistant layer behind, as did extraction with hot water or 0.2 M KOH (SI Fig. S4). In contrast, when 0.1 M HCl was used for extraction, the mild acid completely removed the mucilage layer from the entire seed (SI Fig. S4D), probably hydrolysing it in situ^10^, and in some patches it has also removed the underlying intensely-stained layer (SI Fig. S4F).

### Mucilage accumulation may be independent of embryo and endosperm development

**Figure 5:**
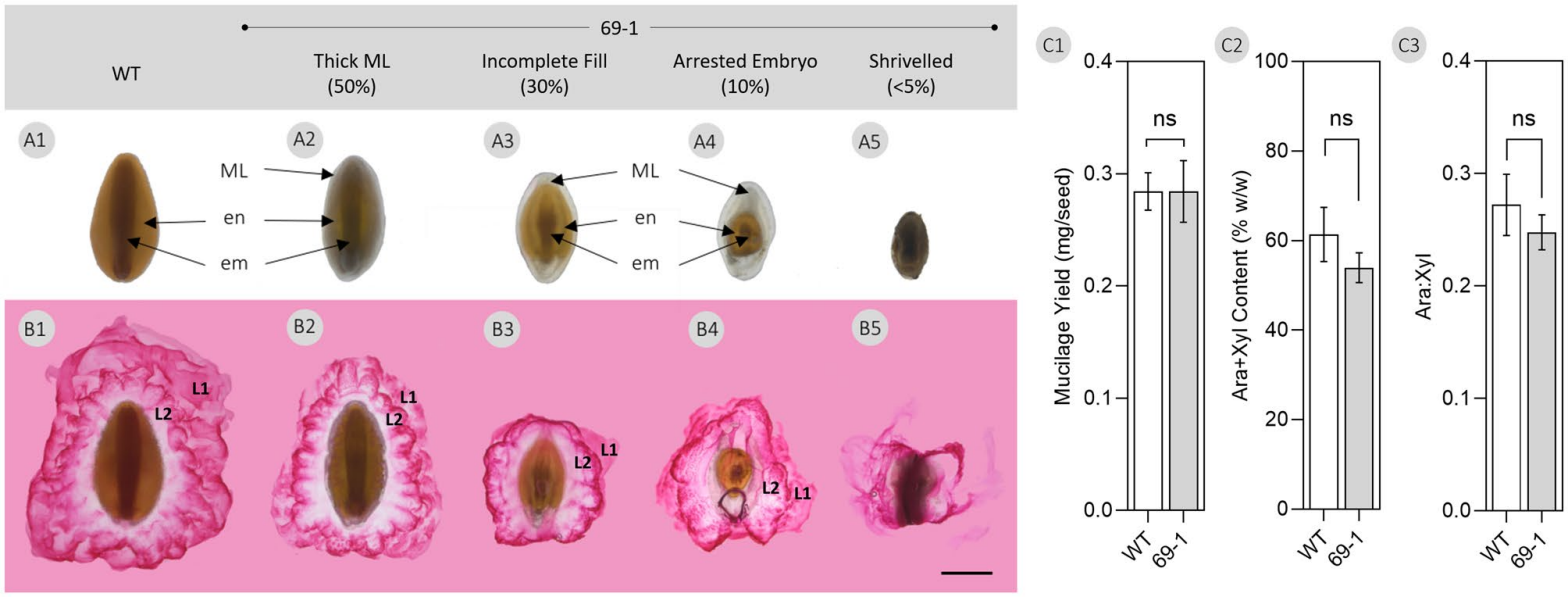
(**A**) Seeds were selected from developmentally-impaired gamma-irradiated *P.* *ovata* mutant 69-1 generated previously by Tucker et al.^43^. When these seeds were imbibed in a ruthenium red solution (0.01% w/v) for 10 min at room temperature (**B**) mucilage expanded from all seeds with different architectures but two typical mucilage layers (L1 and L2) were present. *em* embryo, *en* endosperm, *ML* mucilage layer, *WT* wild-type. Scale bar 1 mm. (**C**) Analysis of mucilage yield and composition revealed no significant difference (ns) from the wild-type (*p* > 0.05, Student’s t test).

From the mutant *P.* *ovata* population reported in Tucker et al.^43^, we selected a line, 69-1, that produces seeds with impaired development across a range of severity: seeds with a thickened translucent outer layer, incomplete endosperm filling, arrested embryo development, or a shrivelled appearance where it was difficult to determine if an embryo was present (Fig. 5A1–5). Ruthenium red staining solution was applied to representative seeds and all types produced mucilage from the seed that was released into the aqueous environment. While different specific architectures were observed, two typical mucilage layers were recognisable in all but the shrivelled phenotype. These seeds may have been aborted early in development rather than representing a developmentally delayed phenotype (Fig. 5B5). While only 5% of seed are WT-like in the 69-1 bulk sample analysed, WT-level total mucilage yields, arabinose and xylan content and ratio were still obtained (Fig. 5C1–3).

### Expansion of seed mucilage polysaccharides across time and space

**Figure 6:**
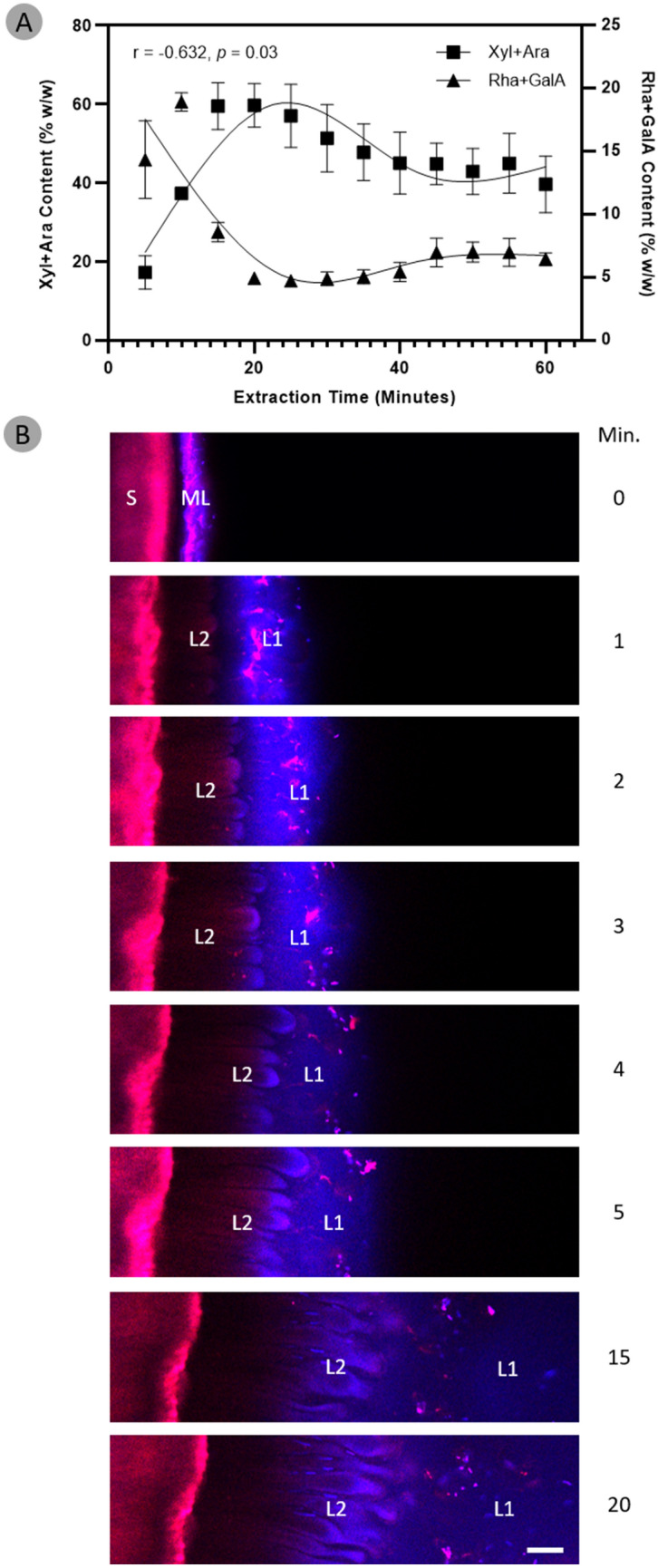
The composition and structure of seed mucilage changes over the course of its expansion. (**A**) Compositional analysis reveals pectin-associated monosaccharides (rhamnose and galacturonic acid) are most abundant during the initial expansion of mucilage and rapidly decrease in concentration thereafter. Heteroxylan-associated monosaccharides (xylose and arabinose) are also present in the initial expansion of mucilage, in almost similar amounts to pectin. Contrasting to pectin, the heteroxylan-associated monosaccharides rapidly increase in concentration and go on to make up the bulk of total expanded mucilage. Data have been fitted with cubic spline curves to highlight trends. (**B**) The dynamics of seed mucilage expansion in mature *P.* *ovata* seeds were observed in real-time over a period of 20 min using confocal microscopy. Seeds were pre-stained with 0.4% Direct Red 23 and 0.1% Calcofluor White. Upon imbibition in water, a sudden “explosion” of an extremely hydrophilic and non-structured mucilage layer emerges (L1). Following L1, a more structured and anemone-like layer of mucilage expands outwards (L2) and by 20 min, L2 has reached its maximal expansion distance and L1 has mostly dissipated into the surrounding aqueous environment. Scale bar 100 μm. *S* mature seed, *ML* mucilage polysaccharide layer on dry seed, *L1* layer 1, *L2* layer 2 N.B. time 0 min is a dry seed that has been pre-stained, water was added after this image was taken.

Microscopy and monosaccharide analysis techniques were used to investigate changes in the composition and structure of the mucilage as it expanded from the seed surface. In order to define temporal mucilage expansion we tried to capture and describe the major stages using chemical profiling. By measuring the release of mucilage-related monosaccharides over 14.5 h we found that the major stages of mucilage expansion occurred within 60 min of imbibition (SI Fig. S5). Monosaccharide analysis of serial fractions taken during the first 60 min of mucilage expansion clearly demonstrated a change in the composition of the expanded seed mucilage over time (Fig. 6A). Relative to total extracted sugars, there was a shift from pectin-dominant to heteroxylan-dominant monosaccharide composition during the initial stages. A sharp increase in the number of heteroxylan-associated monosaccharides (xylose and arabinose) was then observed, peaking at 20 min post imbibition (Fig. 6A) while pectin-derived monosaccharides (rhamnose and galacturonic acid) displayed the inverse, where they were most abundant at the start of seed imbibition and mucilage expansion, before tapering off considerably by 20 min (Fig. 6A).

Mature seeds were stained as described in Yu et al*.*^9^, and real-time mucilage expansion was observed dynamically using confocal microscopy (Fig. 6B). Two distinct layers of expanded mucilage were observed. A third mucilage layer adjacent to the seed coat reported by Yu et al*.*^9^, was not observed. The two mucilage layers, L1 and L2, have distinct structural features. L1 is the first to expand; it lacks clear structure and much of the Calcofluor White staining is associated with this layer. The expansion of L2 from the seed occurs soon after L1 but the sea anemone-like structures (Fig. 6B) are not observed until 2 min post imbibition. By 15 min, L1 has dispersed into the surrounding aqueous environment and by 20 min L2 has expanded in its entirety (Fig. 6B).

## Discussion

**Figure 7:**
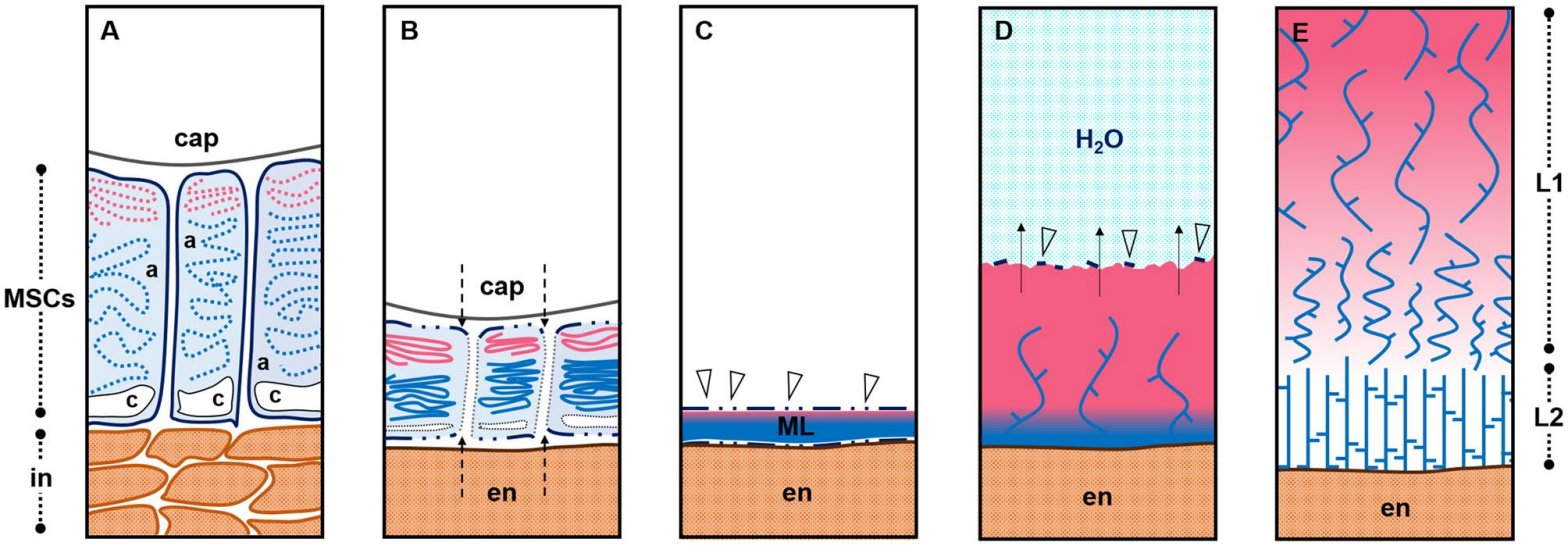
A proposed model of the polysaccharide deposition and mucilage expansion mechanism in *P.* *ovata*. (**A**) Mucilage polysaccharides pectin (pink) and heteroxylan (blue) are polarly synthesised and deposited into the outer apoplast (a) of mucilage secretory cells (MSCs), (**B**) which become compressed between the endosperm (en) and capsule wall (cap), obliterating the radial walls and releasing their contents into a (**C**) continuous laminated cell-free mucilage polysaccharide layer (ML) on the external seed surface when released from the capsule. (**D**) Upon exposure to an aqueous environment, the hydrophilic mucilage starts to expand outwards from the seed surface. The first layer to expand is rich in extremely hydrophilic and soluble pectin and is topped by fragile remnants of the distal MSC walls (arrowheads) that dissolve as hydration continues. This layer works to provide a hydration cascade to initiate and jumpstart hydration and swelling of the more gel-like, less hydrophilic polymers. (**E**) A hypothesised distribution of mucilaginous polysaccharides. The pink gradient is indicative of the distribution of pectin which is restricted to the periphery (or ‘mucilage expansion front’) of the expanded seed mucilage (as per Fig. 6) whilst the xylan polysaccharides (blue strands) are evenly distributed (Fischer et al*.*^12^; Guo et al*.*^55^; Yu et al*.*^9^). In L1, the enrichment of pectin may proportionally modulate the solubility/extractability of xylan while in L2, pectin is not present and thus xylan polymers form a more robust, gel-like layer. *In* integument, *c* cytoplasm.

Mucilage polysaccharide accumulation in the MSCs of *P.* *ovata* seeds follows a different developmental pattern to that occurring in the MSCs of *Arabidopsis*. The mechanism by which different cell layers are converted into seed tissues and the possible remodelling thereafter appears to be of central importance for MSC development in *P.* *ovata*. At some point during mid-development and perhaps after polysaccharide accumulation is complete, we propose that the MSC radial cell walls break down and collapse in a concertina-like fashion. The collapse is potentially driven by the outward pressure of the rapidly expanding embryo and endosperm tissues pushing the MSC outer walls against the inner capsule surface, releasing the accumulated mucilage polysaccharides into an amorphous layer that becomes sandwiched between the remnant distal and basal MSC walls (Fig. 2). The process of radial wall remodelling and/or disintegration may already be beginning by 6/7 DPA where discrete labelling present earlier in development is absent or has become non-specific and amorphous (Fig. 3C1–3,F1–3). This is particularly evident for the CBM3a labelling (Fig. 3F1–3). This is unlike the presence of the mucilage polysaccharides contained within intact discrete cells of the *Arabidopsis* (Fig. 4C,F) and flax^36^ mature seed coats. From late development onwards it is clear that the MSCs of *P.* *ovata* do not contain a columella (Fig. 4B) and our hypothesis is that instead, laminated layers of dehydrated mucilage polysaccharides are present, following radial MSC wall disintegration, between the remnant distal MSC walls, inner capsule wall and the expanded endosperm tissue (Figs. 2, 7B). At seed maturity when released from the dehiscent capsule, the dehydrated and highly compressed mucilage polysaccharides, originating from the obliterated MSC cells, (Figs. 2H, 4H, 7C) form a dense layer over the seed surface (Figs. 2C, 6C). Cross sections of the dry mature seed shows that the thickness of this mucilage layer ranges from 10 to 18 μm compared to the 80 to 90 μm thickness of the MSCs at full elongation at 7 DPA (Fig. 2). This supports the compression of the MSCs as the seed matures, after which point the layer is so dense that the constituent polysaccharides do not label with monoclonal antibodies that bind well to the seed mucilage when expanded (Phan et al*.*^11^; Fig. 2H).

In our SEM analysis of mature seeds, we observe a stark contrast in surface appearance before and after hydration (Fig. 4). After mucilage is hydrated and allowed to dry without fixation, we were no longer able to observe the wrinkled surface or characteristic hexagonal shapes of the underlying distal MSC wall remnants (Fig. 4D–E). We suggest that while the distal walls may have undergone a similar process of remodelling/weakening to the radial walls, they were protected from crushing as they lie perpendicular to the outward force of the expanding endosperm, pushed flat against the inner capsule surface, and thus remain present and visible in the mature seed. However these distal wall fragments are thin, not reinforced with cellulose (Fig. 3F1–3) and appear to be rich in pectin (Fig. 3C1–3) suggesting that they may be highly soluble. We have previously observed ‘hexagonal platelets’ that stain strongly with Ruthenium Red to be released from *P.* *ovata* seeds very early in the hydration cascade and rapidly disintegrate or dissolve (Phan et al*.*^11^, Fig. 1H). We suggest that these structures are the soluble remnants of the distal MSC walls. These are different to other cellulose-staining structures like the mucilage discs of the *Arabidopsis* mutant *fly1*^44^ or plate cells of other *Plantago* species (Phan et al*.*^11^, Fig. 1I–P; Cowley et al*.* unpublished data) which persist through mucilage hydration. When hydrated mucilage is left to dry back onto the seed (Fig. 4D,E), the distal MSC wall fragments may have already dissolved/disintegrated and are thus no longer discernible, so the hexagonal shapes are lost, unlike similarly treated *Arabidopsis* seeds which retain the lower portion of the ruptured MSCs (Fig. 4F). The still soft layer of mucilage polysaccharides released by the putative rupture of the *P.* *ovata* MSC radial walls in later development may also contribute to the polarity of striations on the dorsal side of the mature seed (Fig. 4A). This could occur as the proximal end of the seed is under more compression from the capsule wall, which imprints onto the surface of the polysaccharide layer as it dehydrates. Although the distal end of the seed also comes into contact with the capsule, at this end it does not adhere so tightly and is observed to readily detach when gently touched, thereby enabling seed dispersal. Interestingly, Boesewinkel^45^ suggests that the seed coat of *Linum* *usitassimum* is also polar and that the ‘slime-forming matter’ is deposited on the outer surface of the epidermal seed coat cells. They also suggest that the cells underneath the outer epidermal seed coat cells, which are on the innermost layer of the inner integument, have thickened cell walls and are pigmented cells. These observations are strikingly similar to what we have observed in *P.* *ovata*; the mucilage is the outermost layer of the seed and is underlain by an intensely stained layer that may be pigment-rich (Fig. 2H). A similar structure was also described for *P.* *ovata* by Madgulkar et al.^46^. These observations further support our proposed mechanism of MSC development and disintegration, with the subsequent formation of a cell-free mucilage polysaccharide layer.

It is interesting to speculate about carbon flow through the *P.* *ovata* seed during development. There must be a balance between investment in maternal sporophytic tissue and the filial tissues i.e. the carbon supply must be split between mucilage polysaccharide biosynthesis and feeding the rapidly growing embryo and endosperm. It is possible that development of the MSCs from the outermost cell layer of the integument tissue, which is of maternal origin^31^, could be favoured over zygotic development. This may explain our observation that MSC development and polysaccharide deposition for mucilage synthesis may occur independently of seed development since even developmentally-stalled and aborted seeds still make mucilage when imbibed (Fig. 5B1–5). While the specific architectures were different, two typical mucilage layers were recognisable and known *P.* *ovata* quality indicators^10^, mucilage yield (Fig. 5C1) and heteroxylan content (Fig. 5C2) and composition (Fig. 5C3) were not significantly different to the wild-type (*p* > 0.05) showing that mucilage synthesis was uninterrupted. Garcia et al.^47^, demonstrated that development of the maternally-derived integument and the zygotic embryo and endosperm are coordinated to determine final *Arabidopsis* seed size. Of the various developmentally-impaired *P.* *ovata* seeds analysed here, none of them reached the same size as the wild-type, suggesting that although integument development and mucilage polysaccharide biosynthesis can occur independently of embryo and endosperm development, some coordination is needed in order to establish the correct size of the mature seed. It is possible that without outward pressure from the growing endosperm not only will the seed not reach mature size, but the MSC contents may not be correctly arranged and/or pressurised causing the diminished mucilage expansion shown here. Unlike *Arabidopsis*, several *Plantago* species are reported to contain specialised nutrient transfer structures called haustoria that develop from the embryo sac. Haustoria have been characterised in the seeds of *P.* *lanceolata*^48^, *P.* *major*^49^, and *P.* *coronopus*, while those in *P.* *pumila* (also known as *P.* *exigua*) and *P.* *lagopus* are described briefly by Johri et al.^50^. Cooper^48^ observed haustoria “penetrating and digesting the outer portion of the ovule adjacent to the developing endosperm”, in *P.* *lanceolata*, and this corresponds to the layer we have designated integument in *P.* *ovata*. Haustoria may function to directly connect the embryo and endosperm to surrounding integument cells, allowing a networked supply of carbon for growth and development. Eventually, the growing endosperm of *P.* *lanceolata* absorbs most of the surrounding integument and leaves only a few cell layers that lose most of their cytoplasmic contents and are squashed thin at maturity. Although Cooper^48^ did not specifically state what this papery-thin layer could be, it is likely that they were describing the mucilage polysaccharide layer. Haustoria have not yet been reported in *P.* *ovata*, and we were unable to confirm their presence or absence in the developmental sections presented here. Thus, it remains unclear what mechanisms control the fate of the integument cells and this will be informative to investigate in the future.

The microscopy images of the developing MSCs (Fig. 2) raise questions regarding gene expression and regulation during the different developmental stages. During the early stages of MSC development at 3–5 DPA where rapid cell expansion is observed, the enzymes that are present may be synthesising the backbone of the immature pectin polymer i.e. one that still requires post-synthesis modification to become hydrophilic, as observed in *Arabidopsis*^51–53^ and early stages of heteroxylan synthesis may also be occurring. The shift in the esterification status of the pectin in both the MSCs and the integument cell walls is clearly demonstrated in Fig. 3 and SI Fig. S2, where tissues at anthesis label differently when compared to four days later. However, at this magnification it is not possible to unequivocally define the location of the pectin—whether it is in the actual wall of the MSCs or in the apoplast and just pushed tightly against it will require detailed examination at the TEM level. There is a clear increase in the amount of crystalline cellulose in the walls of both the MSCs and the integument cells when tissues at anthesis and later at 4 and 7 DPA are compared. At these early stages there is minimal binding by the LM11 antibody which detects xylan suggesting there is no xylan present yet, even in the cell walls rather than the apoplast (SI Fig. S2B,D,F). This lack of signal is consistent with our previous analyses, where later in development (13 DPA) many of the genes involved in xylan biosynthesis are transcriptionally active, such as the GT61, UXS, and UAM genes^11^ which is only two days before the 15 DPA stage when the MSCs appear misshapen and possibly on the verge of extensive disintegration (Fig. 2G). It is possible that at 13 DPA these genes are more involved in polysaccharide post-synthesis modification, modifying the sidechain density and/or length in xylan and pectic polymers to enable correct mucilage expansion and final architecture upon imbibition in aqueous environments, but at this stage details are unknown. Future experiments are aimed at establishing the transcript abundance of gene sub-sets involved in synthesis of the pectic backbone including GT8, GAUT1 and GAUT7^54^ members, the addition of minor substituents onto the pectin backbone and the biosynthesis of nascent and mature heteroxylan types. A precise temporal series employing laser capture microdissection of the developing MSCs, followed by RNAseq analysis will prove invaluable in this context.

Our microscopy analyses reveal that, in contrast to *Arabidopsis*, the seed mucilage release mechanism of *P.* *ovata* may be a physical process not dependent on cellular rupture followed by extrusion (Fig. 6B). Hence, for this species we suggest that it is more appropriate to describe the mechanism as an expansion rather than an extrusion, similar to the extension of a concertina, where the mucilage starts to expand into the aqueous environment as it hydrates from the wrinkled appressed polysaccharide layer on the seed surface. Data demonstrating the change in mucilage composition and structure over time (Fig. 6) support this type of expansion process. The driving forces behind expansion of *P.* *ovata* seed mucilage could be derived from the differential hydrophilicity of the constituent mucilaginous polysaccharides. Pectin is most abundant in the first layer of mucilage to expand, L1 (Fig. 6B) and previous studies have described the outermost layer of the expanded seed mucilage to be a highly soluble, pectin-rich fraction^9,12,55^. In *P.* *ovata*, this seed mucilage fraction was easily extracted using cold water^9,10^ and we propose that its function is to act as a primer, initiating mucilage expansion, and providing a hydration cascade triggering the swelling of the more gel-like and structurally-complex heteroxylan polymers located in subsequent fractions/layers (SI Fig. S6). Compositional data supporting such patterns of polymer release have been reported previously^9,10,13^, and now a model to illustrate this process, driven by polarised deposition and then expansion, is presented in Fig. 7. To fulfil such a role the pectin-enriched fraction must be synthesised and/or deposited first into the distal end of the MSC, anchoring the mucilage to the seed to form L1, after which the structural polymers, including heteroxylan, are synthesised and/or deposited into the basal end of the cell to make L2 (Fig. 6A). A similar spatio-temporal pattern of polysaccharide synthesis and deposition was recently described by Miart et al*.*^36^ who showed that RG-I (pectin) was synthesised in the two outermost layers of *L.* *usitatissimum* MSCs prior to synthesis of other polysaccharides in the layers beneath. The authors hypothesised that the arabinoxylan, xyloglucan and cellulose polysaccharides synthesised later in the inner layers provided a structural element that pressurised the outermost contents, enabling efficient mucilage release and anchoring the mucilage to the seed. Similarly, the heterotypic interactions of various polymers including branched xylan, cellulose and arabinogalactan proteins are important for effective mucilage release and adherence in *Arabidopsis*^3,5,6,8,19,56^. In support of this temporal sequence of events, real-time qPCR analysis of cDNA from developing *L.* *usitatissimum* integument tissue shows that genes involved in pectin biosynthesis are transcriptionally active prior to those associated with xylan biosynthesis (Aubert et al., University of Adelaide, unpublished data). The mucilage component of *P.* *ovata*, and likely many other species, is a complex network of heterogeneously distributed polysaccharides. Each polymer must be synthesised, deposited, and potentially modified, in a specific sequence and location during seed development to be able to fulfil the required mechanical functions enabling mucilage release, and supporting the structural functions of the material once it extends from the seed surface. The process of mucilage polysaccharide biosynthesis and deposition into the MSCs must therefore be a tightly regulated process, about which we have much to learn.

In future work, it would be valuable to characterise the MSCs of *Plantago* species such as *P.* *cunninghamii* that we have already confirmed to possess a similar seed surface arrangement to *P.* *ovata*, but that has a different expanded mucilage architecture^11^. Our preliminary characterisation of the MSCs of *P.* *ovata* has generated further questions regarding the biosynthesis and deposition of mucilaginous polysaccharides: how, where, and in what order are these polymers transported and deposited into the MSCs? What genes and regulatory elements are controlling this highly complex process? And what drives the fate of the integument cells? Should we be looking for signs of programmed cell death or a suite of cell wall degrading enzymes in this tissue? Combining further histological analysis of the developing seeds, with a focus on the MSCs and the integument tissue, with characterisation of the temporal regulation of mucilage biosynthetic transcripts may begin to answer some of these questions. Our whole mount immunolabelling data suggests that hydrated heteroxylan is distributed in a specific digit-like pattern whilst immunolabelling of seed sections show that the heteroxylan and pectin are homogenously distributed throughout the expanded seed mucilage (Phan et al*.*^11^). It remains unclear how these polymers are deposited and distributed in both the developing MSCs, the mature mucilage polysaccharide layer, and the final expanded material. Thorough investigation of the developing MSCs in *P.* *ovata* may begin to shed some light upon these questions and allow us to fine tune our hypothetical model, whilst eventually providing tools to allow manipulation of the mucilage quantity and quality that could directly impact downstream applications and economics of psyllium use.

## Materials and methods

### Plant materials and growth

Wild-type *P. ovata* and gamma-irradiated *P.* *ovata* mutant 69-1 were obtained from a population previously generated by Tucker et al*.*^43^. Three 69-1 sister lines at M4 were tested to show that > 95% of the seeds in each sister line displayed a developmentally delayed phenotype. The mutant line has not yet been backcrossed to wild type.

Plants were grown as per Phan et al*.*^11^*.* To stage wild-type fruit development, fruits with freshly emerged anthers (erect and bright-yellow in colour) were marked and tagged with the date in order to harvest at the relevant day post-anthesis (DPA).

### Observing *P. ovata* expanded seed mucilage

#### Ruthenium red

Mature *P.* *ovata* seeds were individually placed onto microscopy slides in a ruthenium red solution at a concentration of 0.01% (w/v) (ProSciTech, C075, Australia). Seeds were observed under a Zeiss Stemi 2000-C dissecting microscope with an attached AxioCam ERc 5s camera. Seeds with impaired development were selected from the mutant line 69-1^43^.

#### Time-lapse

Mature *P.* *ovata* seeds were prepared as per Yu et al.^9^. In brief, dry mature *P.* *ovata* seeds were soaked overnight in stain solution comprised of 0.1% w/v Calcofluor White (Fluorescent Brightener 28, Sigma-Aldrich) and 0.4% w/v Direct Red 23 (Sigma-Aldrich) diluted in 80% ethanol. The seeds were removed from the staining solution and allowed to air dry before being adhered with a cyanoacrylate adhesive to the centre of a Petri dish. The Petri dish was mounted onto the stage of a Nikon A1R Laser Scanning Confocal with DS-Ri1 CCD camera and imaged prior to the addition of deionised water onto the seed at time = 0. Images were captured for 20 min in total at 1 min intervals.

### Fixation, embedding, and sectioning of *P. ovata* developing fruit

Samples requiring fixation, embedding, and sectioning were processed as per Burton et al.^57^ and embedded tissue was sectioned at 1 μm on an Ultramicrotome (Leica, EM UC6) using a diamond knife (DiATOME, Nidau, Switzerland). For non-aqueous fixation, PBS was replaced with an 80% ethanol solution. Sections were stained with Toluidine Blue (epoxy tissue stain, used undiluted, ProSciTech, C149, Australia). Sections were imaged under transmitted light differential interference contrast (DIC) using a Zeiss Axio Imager M2 (Carl Zeiss, Germany) fitted with an AxioCam MRm3 monochrome camera.

For fluorescence images, samples were fixed, embedded, and sectioned as above for non-aqueous fixation. Survey sections were stained with epoxy tissue stain (used undiluted, ProSciTech, Australia). For immunofluorescence, sections were incubated with monoclonal antibodies raised against pectin (LM19 and LM20) and arabinoxylan (LM11) (PlantProbes Leeds, UK) followed by an appropriate AlexaFluor 555 secondary antibody (Invitrogen, USA). The His-tagged carbohydrate-binding module CBM3a (PlantProbes, Leeds, UK) was used with a triple indirect immunofluorescence labelling procedure as described previously^11,58^. Sections were counterstained using Calcofluor White (Fluorescent Brightener 28; Sigma-Aldrich) and mounted in glycerol. Images were obtained using an AxioCam 105 color camera fitted to a Zeiss fluorescence microscope (Axio Imager M2, Carl Zeiss, Germany) with 254/432 nm excitation/emission wavelength for Calcofluor White and 553/568 nm excitation/emission wavelength for LM11/LM19/LM20/CBM3a.

### Scanning electron microscopy

Mature seeds of *P.* *ovata* and *Arabidopsis* *thaliana* ecotype *Columbia-0* were air-dried before and after mucilage hydration then sputter-coated with platinum at a thickness of 5 nm. Seeds were imaged at a working distance of 7.5 mm with an accelerating voltage of 3 kV using a Philips XL20 Scanning Electron Microscope (SEM) following Phan et al*.*^11^.

### Mucilage extraction and compositional analysis

For temporal mucilage analysis, mucilage was collected by placing 1 g mature seeds into a wide-mouth sieve, placed in a water bath containing 40 mL of deionised water at room temperature with intermittent stirring. Fractions were collected at 5 min intervals by transferring the sieve and seeds into a fresh batch of deionised water. Mucilage extracts were freeze–dried to a constant weight and compositional analysis was conducted as per Hassan et al*.*^59^.

For comparison of mutant 69-1 with the wild-type, mucilage was extracted from 40 seeds for 3 h in 20 mL of deionised water heated to 80 °C and stirred vigorously on a heated magnetic stirrer. While still hot, the mucilage and seeds were transferred into a 50 mL tube and centrifuged for 10 min at 4,000 rpm. The mucilage supernatant was decanted into a new tube and freeze–dried to a constant weight. Yield per seed was calculated by dividing the freeze–dried mucilage mass by 40. Compositional analysis was conducted as per Hassan et al*.*^59^.

## Supplementary information

Supplementary file1 (DOCX 4695 kb)

## Data Availability

The datasets generated and/or analysed in this study are available from the corresponding author on reasonable request.

## Abbreviations

DPA: Days post-anthesis
ML: Mucilage layer
MSC: Mucilage secretory cell
SEM: Scanning electron microscopy
