# Supplementary material for: The novel features of Plantagoovata seed mucilage accumulation, storage and release
Source: Sci Rep. 2020 Jul 16;10:11766. doi: 10.1038/s41598-020-68685-w (PMC7366641; doi:10.1038/s41598-020-68685-w)
Supplement: Supplementary file 1 — Supplementary file1 (DOCX 4695 kb) [file 41598_2020_68685_MOESM1_ESM.docx]

**Title:**

The novel features of *Plantago* *ovata* seed mucilage accumulation, storage and release

**Authors:**

Jana L. Phan^1#†^, James M. Cowley^1,2#^, Kylie A. Neumann^1,2‡^, Lina Herliana^2^, Lisa A. O’Donovan^2^ and Rachel A. Burton^1,2^*

[jana.phan@science.org.au](mailto:jana.phan@science.org.au)
[james.cowley@adelaide.edu.au](mailto:james.cowley@adelaide.edu.au)
[kylie.neumann@ipaustralia.gov.au](mailto:kylie.neumann@ipaustralia.gov.au)
[lina.herliana@adelaide.edu.au](mailto:lina.herliana@adelaide.edu.au)
[lisa.odonovan@adelaide.edu.au](mailto:lisa.odonovan@adelaide.edu.au)

*Corresponding author, [rachel.burton@adelaide.edu.au](mailto:rachel.burton@adelaide.edu.au), +61 08 8313 6501

# These authors contributed equally

^1^Australian Research Council Centre of Excellence in Plant Cell Walls, School of Agriculture, Food and Wine, University of Adelaide, Waite Campus, Urrbrae, SA 5064, Australia

^2^Australian Research Council Centre of Excellence in Plant Energy Biology, School of Agriculture, Food and Wine, University of Adelaide, Waite Campus, Urrbrae, SA 5064, Australia

**
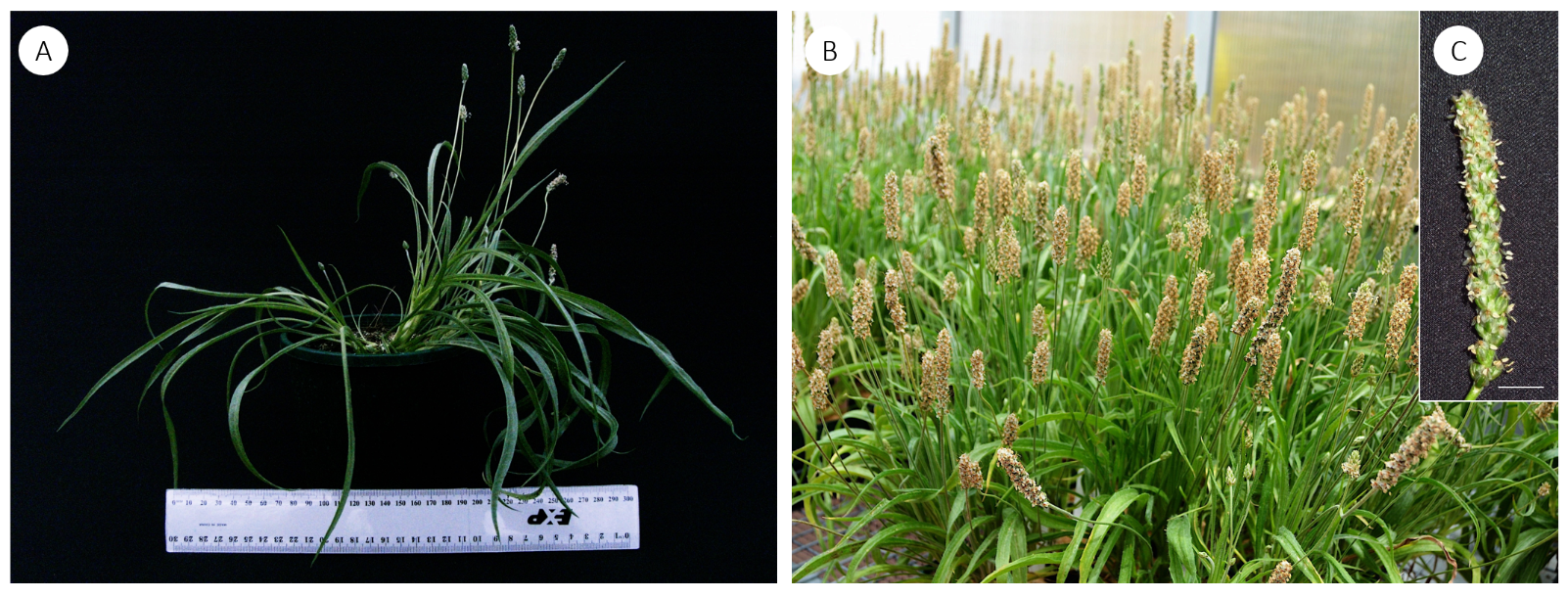
**

**Supplementary Fig. S1.** *Plantago ovata* grown in a glass house. **(A)** shows a 2.5-month-old plant (ruler = 30 cm) and **(B)** shows 3.5-month-old plants with fully set inflorescences where the seed heads are almost completely dry and ready for harvesting. **(C)** shows a single inflorescence at ~3 months old, scale = 1 cm.

**
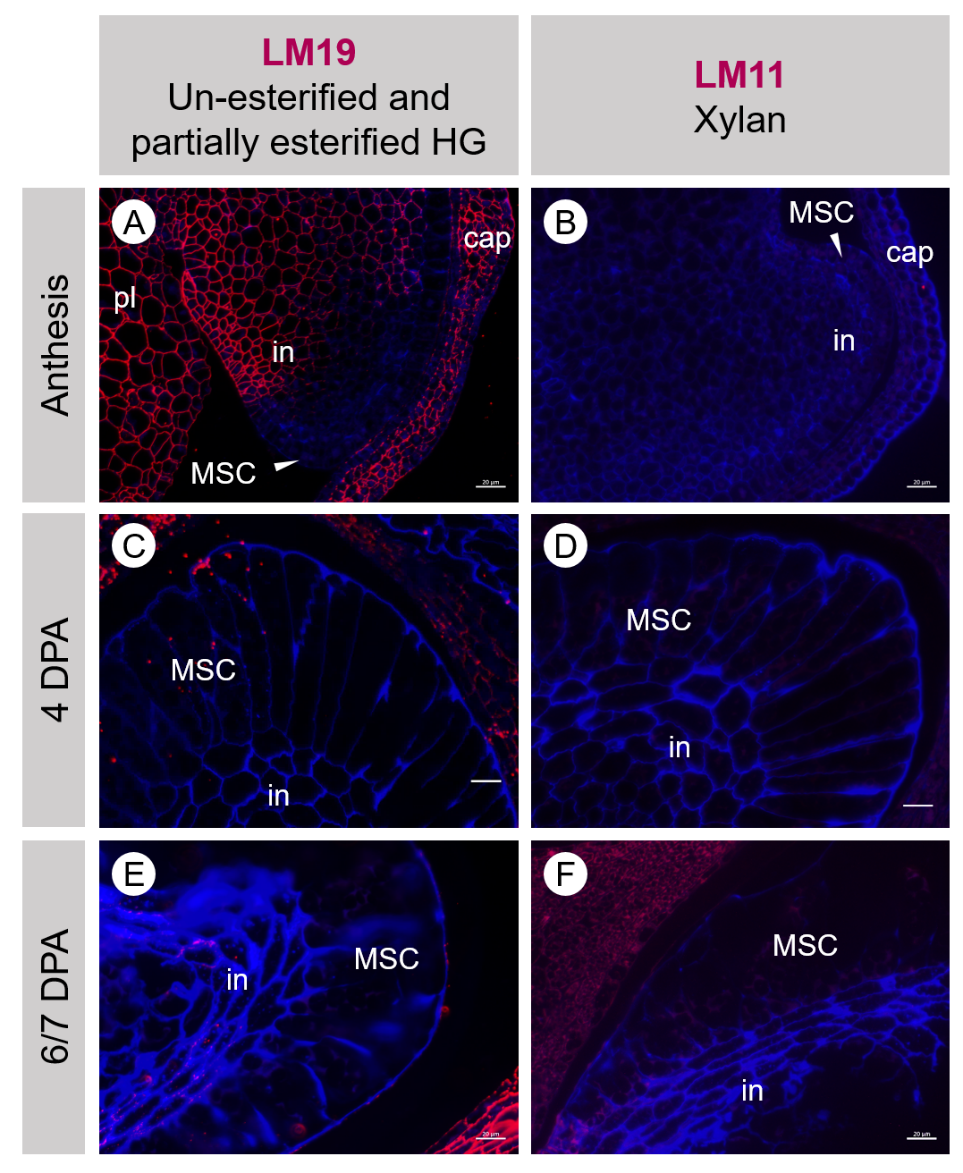
**

**Supplementary Fig. S2.** From anthesis to the beginning of mucilage secretory cell (MSC) disintegration, minimal labelling of un-esterified/partially esterified homogalacturonan (HG) (LM19) and β-1,4-linked xylan backbone (LM11) is observed in developing seed tissues. Scale bar = 20 μm; MSC = mucilage secretory cell; in = integument.


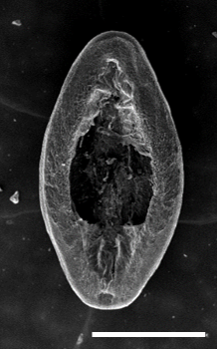


**Supplementary Fig. S3.** The mature seed of *P. ovata* has a deep scar on the proximal side**,** a remnant of where the seed was attached to the placental tissue and now forms the cymbiform shape of the seed.


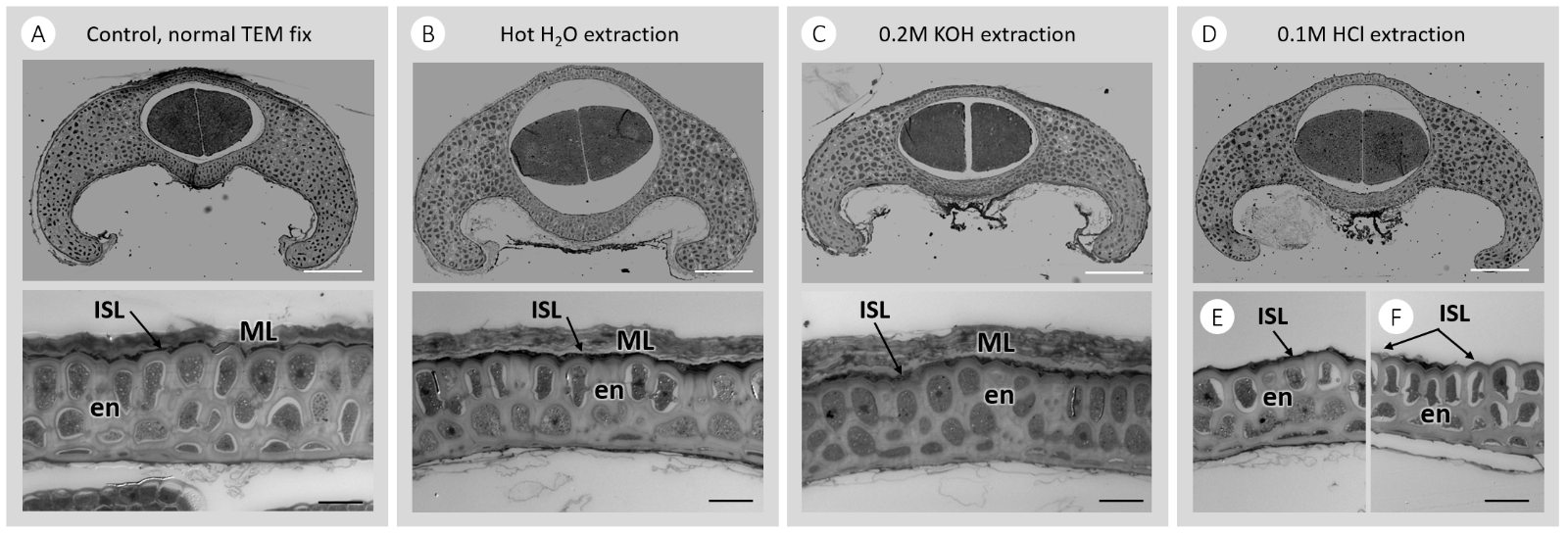


**Supplementary Fig. S4.** Toluidine blue-stained transverse sections of mature *P. ovata* seeds after different methods of mucilage extraction. The expanded seed mucilage has been removed from the control **(A)** during the sequential washing steps required in tissue fixation, some mucilage remains tightly adhered to the seed after hot water extraction **(B)** or treatment with 0.2M KOH **(C).** Treatment with 0.1M HCl removed all the mucilage material **(D)** and **(E)** plus some sections of the intensely stained layer **(F).** All extractions were performed at 60°C for 3 hr on a magnetic stirrer. en = endosperm; ML = mucilage layer; ISL = intensely stained layer. Bars for whole seed = 200 μm, and the endosperm and mucilage layer = 20 μm.


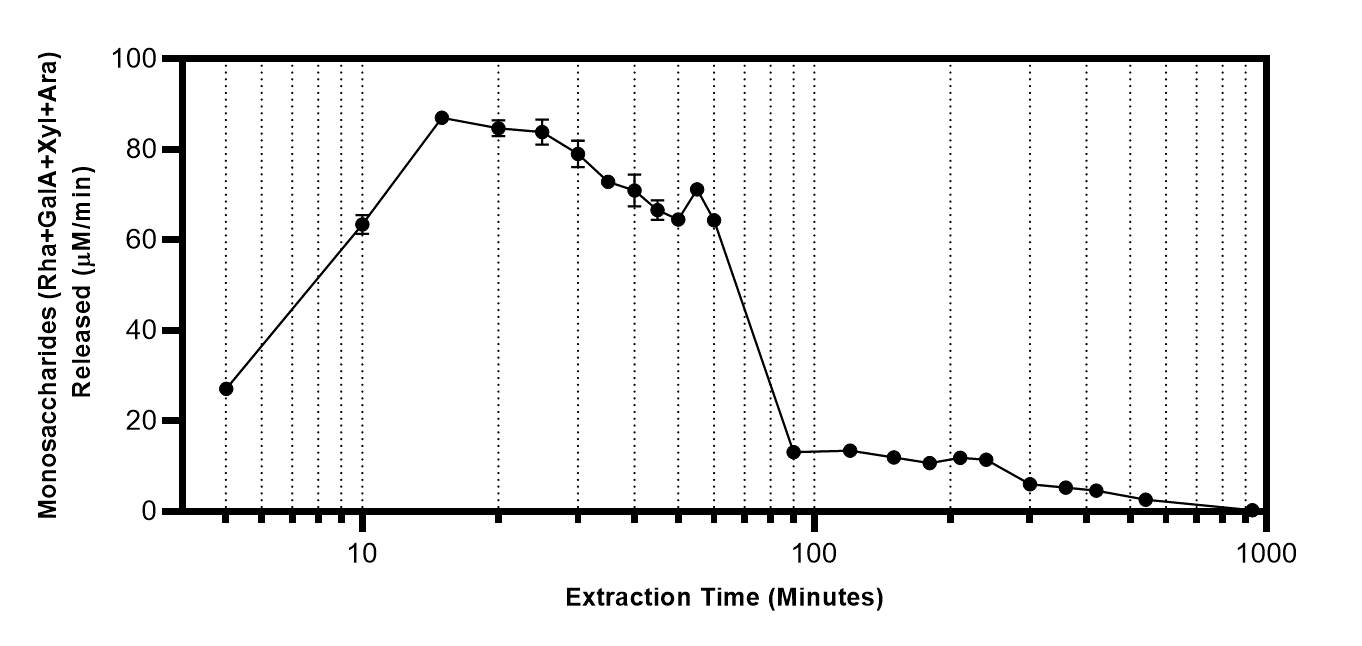


**Supplementary Fig. S5**. Extraction of mucilage up to 60 mins from the start of imbibition effectively captures all major stages of *P. ovata* mucilage release. Semi-log plot shows that mucilage-related monosaccharide release tapers off sharply after 60 mins and only small quantities of monosaccharides are released in the subsequent 14.5 hours (870 mins).


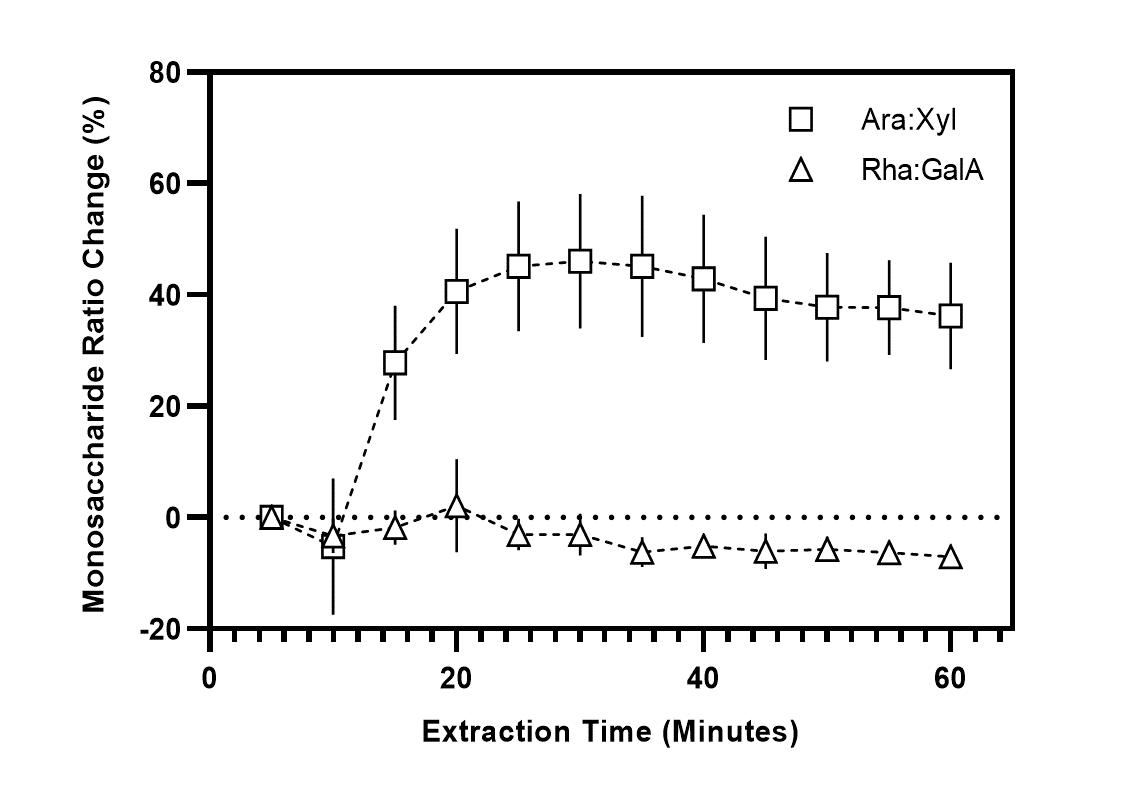


**Supplementary Fig. S6**. While the pectin Rha:GalA ratio remains relatively unchanged between sequentially isolated fractions, the Ara:Xyl ratio gradually increases, suggesting increasing polysaccharide complexity through time. The monosaccharide ratio difference at an extraction time-point is calculated relative to the initial monosaccharide ratio at 5 min i.e. (initial ratio, 5 min / ratio at a timepoint, x min).
